# Disease in a Debt Crisis: Financing Global Health, Development and AIDS between WHO and World Bank, 1978–87

**DOI:** 10.1017/mdh.2020.17

**Published:** 2020-07

**Authors:** Reiko Kanazawa

**Affiliations:** Centre for Biomedicine, Self and Society, 23 Buccleuch Place, University of Edinburgh, UK

**Keywords:** Global health, Structural adjustment, Neoliberalism, Health systems financing, HIV/AIDS

## Abstract

This article examines how international organisations with mandates in health and development interpret global economic crises and respond to disease. It contributes the perspective of World Bank to emerging scholarship on the various factors leading to the decline of the World Health Organization (WHO) and its Health for All (HFA) mission during structural adjustment. It does so by telling a story of collaboration and conflict between WHO and World Bank’s Population, Health and Nutrition (PHN) Department following the ambitious Alma Ata Declaration in 1978 until the initial global AIDS response. As debt crises emerged in Latin America in the early 1980s, WHO tried to find a way forward for HFA. However, the African crisis of 1985 fractured the international community’s support, causing WHO and PHN to dialogue more closely regarding health sector financing. As AIDS became a global crisis, this culminated in their 1987 joint research on the disease’s macroeconomic and demographic impact. However, observing WHO’s continued hesitance regarding financing and its decision to act as a donor gatekeeper, the Bank ultimately opted to work separately in AIDS. Thus, the themes of the Alma Ata versus Selective Primary Health Care debate of the late 1970s continued throughout the 1980s into the early years of the global AIDS response: a perennial conflict of financing within resource constraints and the appropriate role of donors in the grand project of health and development.

## 1 Introduction

In the wake of the 2008 global financial crisis, the World Health Organization (WHO) once again reoriented its focus towards the social determinants of health.^1^ Hearkening back to the Alma Ata Conference on Primary Health Care, Director-General Margaret Chan declared that:

[a] previous effort to use health as the route to socioeconomic development, launched in 1978, was followed almost immediately by a fuel crisis, soaring oil prices, and the debt crises of the early 1980s. In the international response to these crises, mistakes were made when budgets were shifted away from investments in the social sectors, most notably health and education.^2^

The incident referred to was the aftermath of WHO’s call for Health for All (HFA) and the rise of the World Bank’s neoliberal stance on health financing in the 1980s, which historians have characterised as the transition from ‘international’ to ‘global health’.^3^ In the immediate post-war period, WHO pursued technocentric disease programmes with varying degrees of success. By the liberal 1970s and the rise of the non-aligned movement, WHO’s social medicine-oriented Director-General Halfdan Mahler famously declared commitment to Primary Health Care (PHC) in 1978. Similarly, though World Bank was intended to reconstruct a war-torn Europe, in the 1970s under the leadership of President Robert McNamara, it turned attention to social sector issues first in population and later in nutrition, health and disease, as Jennifer Ruger has detailed.^4^ This culminated in McNamara’s establishment of the Population, Health and Nutrition (PHN) Department in October 1979, which allowed the Bank to loan for single-issue health projects. Unlike the UN system, the Bank raises funds through private markets or trust funds, and could thus issue project loans at scales WHO’s grant-funded programmes could not. Finally, as the home of the International Monetary Fund (IMF), the lender of last resort for national balance of payments crises, the Bank was integrated into the international financial system in ways that WHO was not.

The Bank’s role in structural adjustment during the 1980s has been controversial. This was because of the global economic recession in developing countries following the 1979 oil shock, the impact of which World Bank had a significant hand in managing. Critical public health scholarship has argued that the Bank through its various instruments set loan conditionalities that restructured the role of the state in delivering public goods such as health.^5^ It has shown that, in contrast to HFA, the reforms reduced public health in developing countries to focus solely on diseases that represented the greatest burden, extending from the perspective of Selective Primary Health Care (SPHC). SPHC presumed that in contexts of severe resource constraints, developing countries could do little more than control the worst of infectious diseases, echoing colonial public health wherein the main concern was the security and protection of empire. Operationally, this view was expressed through vertical targeted interventions for single diseases that represented the most cost, direct or indirect, run by a mix of public, non-profit and for-profit private actors.

Histories of international and global health have since pointed to a wide range of factors that led to the decline of WHO and its Health for All mission. Earlier scholarship focused on Cold War politics, such as the fight to hold the 1978 conference in the Kazakh Soviet Socialist Republic, which Socrates Litsios has illuminated.^6^ Alexander Medcalf and João Nunes analysed WHO’s visual mass communication strategies to ‘market’ HFA.^7^ Marcos Cueto and Randall Packard have pointed to former WHO supporters such as UNICEF deciding cost-effective interventions for children would be more realistic, particularly immunisations, breastfeeding and oral rehydration.^8^ William Muraskin has shown the subsequent resurgence of eradication fascination to replace HFA.^9^ Finally, Marcos Cueto, Theodore Brown and Elizabeth Fee show that HFA faced particular challenges under Mahler’s successor, Hiroshi Nakajima, who focused on the biomedical aspects of health at the expense of primary care and personally displayed a lack of diplomacy with the US and health funders of increasing influence like the Bank.^10^

Scholars have also focused on how the broader 1980s debt crises impacted international organisations’ perceptions of their respective missions in the health field. Nitsan Chorev identified the United States under President Ronald Reagan as the main reason for the neoliberal shift in international health.^11^ Reagan sought to combat what was perceived as overrepresentation of developing countries’ interests at UN forums, including WHO’s primary health care, and sought instead to work through World Bank to ensure developing countries repaid their loans to American banks. Michael Merson and Stephen Inrig recently recounted how in the early years of the Global Programme on AIDS, Director Jonathan Mann was ambivalent towards the Bank’s push for cost-effectiveness in AIDS interventions.^12^

This article contributes to this emerging scholarly strand on the ‘neoliberal turn’ in global health. By drawing on archival material from World Bank, it incorporates the perspective of Bank health experts in the PHN Department from the aftermath of Alma Ata throughout the 1980s, at the same time that AIDS emerged as an unknown yet potentially devastating disease. It sheds new light on the negotiations, collaborations and disagreements between the two agencies, filling a gap in the recent historiography that seeks to unearth the various interlocking factors for HFA’s decline. The article suggests that the new neoliberal logic in global health was co-produced through WHO and PHN’s differing interpretations of the 1980s debt crises, as well as their contrasting views on the appropriate role of international agencies in health and development.

The relationship between WHO and PHN was complex and interdependent during structural adjustment, as the lines between health and development blurred. Both institutions were concerned about the health sector austerity measures that the IMF and the International Bank for Reconstruction and Development (IBRD) conditional loans dictated and HFA was a powerful statement that provided a common framework, until the mid-1980s. However, whenever they did collaborate, the two were each other’s harshest critics. PHN thought WHO was making promises it would inevitably be unable to fulfill, and its response to the 1985 African crisis of famine, civil wars and debt seemed to confirm this view. Conversely, WHO tended to be stand-offish and aloof, and did not make as much effort to understand PHN or the Bank’s approach. This was rooted in WHO’s sensitivity to the Bank’s growing influence in health, as Jason Finkle and Barbara Crane have shown regarding their population control work in the 1960s and 1970s.^13^

The article proceeds as follows. First, it looks at how WHO and PHN both re-strategised their health work amidst balance of payments crises emerging in Latin American countries in the early 1980s. Second, it shows that the African crisis of 1985 was a critical test for the logic underpinning HFA, ushering in the era of health systems financing. Third, it examines PHN and WHO’s 1987 collaboration for AIDS as the culmination of their discussions on financing. Finally, it studies the separate actions WHO and the Bank took in the early global AIDS response, selectively incorporating their ongoing dialogue with one another. In doing so, this article situates global health in the 1980s within broader narratives of international development and financing, making a case for histories that relate complex interactions between multiple actors.^14^

## 2 Debt Crises after Alma Ata vs Selective Primary Health Care

Although WHO and its Constitution established on 7 April 1948 that health was ‘a state of complete physical, mental, and social well-being and not merely the absence of disease or infirmity’, the UN body initially pursued technocentric ‘magic-bullet’ interventions for malaria, tuberculosis and smallpox.^15^ However, questions of global equity, social justice and redistribution of resources were gaining momentum during the 1960s and 1970s through the non-aligned movement and its vision for a New International Economic Order (NIEO). As Nils Gilman has detailed, NIEO in its various iterations laid out a radical programme for action that would do away with the persistence of colonial economic hegemony and integrate developing countries as full and equal participants in the international community.^16^

It was within this broader climate, as well as facing criticism of its vertical disease control programmes, that WHO Director-General Halfdan Mahler famously declared ‘Health for All by the Year 2000’ at the 1978 International Conference on Primary Health Care in Alma Ata, Kazakhstan.^17^ Mahler had extensive experience integrating tuberculosis control in basic health systems and would be HFA’s staunchest advocate. From sanitation and safe drinking water to maternal and child health, Alma Ata ambitiously proposed a holistic bottom-up approach to the organisation of health sectors. As Chorev has shown, WHO was also taking a stance on development aid relationships, as Alma Ata was particularly encouraging of the flow of technical assistance as well as financial support from developed to developing countries.^18^ The Declaration argued that all nations should ‘cooperate in a spirit of partnership and service to ensure primary health care for all people since the attainment of health by people in any one country directly concerns and benefits every other country’.^19^ In addition, the Thirty-second World Health Assembly the following year placed particular emphasis on the role WHO should play as coordinator for financial, technical and educational resources for health and to this end, a health resources mobilisation group was constituted.^20^ Thus, a significant component of WHO’s HFA was the vision for a radically restructured global political economy and a specific mode of technological and financial transfers for health that would flow through WHO.^21^

Historians have recounted how HFA was subsequently critiqued for being vague, overambitious and failing to take into account limited resources. James Grant, executive director of UNICEF, though sympathetic to primary care and once WHO’s ally in the Alma Ata declaration, joined Robert MacNamara of World Bank, John Gillian of USAID and John Knowles of Rockefeller Foundation at the Bellagio Conference in 1979 to counter-propose with the approach of Selective Primary Health Care (SPHC).^22^ SPHC was based on Julia Walsh and Kenneth Warren’s controversial paper arguing for more cost-effective, focused and practical alternatives towards the desirable but currently unattainable goal of comprehensive primary care.^23^ It evaluated the major diseases of developing countries according to prevalence, mortality, morbidity and feasibility of control. How high a disease was ranked would determine the magnitude of its ‘burden’ on the nation and the point of intervention, which had to then be carefully planned to ‘use the limited human and financial resources available most effectively and efficiently’.^24^

When Mahler announced HFA, Bank health experts had misgivings primarily because it signalled that WHO’s mandate had been overhauled. From being ‘one of the foremost professionally competent organisations’ under the previous Director General, Marcolino Gomes Candau, that once housed experts on all infectious diseases and served as the centre of international health regulations, PHN staff felt Mahler was announcing WHO as a developmental organisation for social justice.^25^ Nevertheless, it is key to note that the Bank was supportive of HFA in the early 1980s. First was that HFA was a powerful statement with much popular support. As health was a still developing area competing with its more traditional work in population and nutrition, the Bank was still in the position of learning from WHO. Bernhard Liese, formerly an assistant for public health in the Operations Policy Department and later deputy chief of PHN in 1980, recalled that ‘[Alma Ata] was actually in that sense very important because it showed to the two of us [Liese and colleague K. Kanagaratnam of the Population Projects Department] very clearly – and we related that to senior management – that we either be part of primary care or we would not be active in the health sector’.^26^ As a result, Bank health experts positioned themselves as ‘mediators’ between HFA and Rockefeller’s controversial Walsh/Warren paper.

In the Bank, we were sitting somewhere on the fence on this discussion. We were sitting between WHO and the Rockefeller Foundation. And what was our role? Our role was basically to keep the communication open on both sides.^27^

Furthermore, HFA’s low-cost, simple and preventive focus seemed to confirm the ongoing basic needs trajectory under McNamara.^28^ The Bank’s expansion into health resulted partially from critiques of the environmental impact of its development projects throughout the 1960s and 1970s, particularly dams. As environmental advisor James Lee (who initiated the WHO–UNDP–World Bank Tropical Diseases Research Programme) stated, the Bank began considering schistosomiasis, onchocerciasis, malaria and other disease prevention in projects dealing with irrigation and water use from the mid-1970s.^29^ Thus, when the Executive Board approved PHN to make loans for health in July 1979, Liese recalled: ‘[T]here was no opposition. There were questions, but it sailed through wonderfully, as it had been something which was overdue.’^30^ Finally, the privatisation of American healthcare was seen as an example to avoid, as the Bank’s first 1975 *Health Sector Policy Paper* cautioned against technologised and expensive tertiary care.^31^

The tone of overall support for HFA with some reservations is perfectly encapsulated in the PHN’s first output in 1980, setting the tone for the Bank’s first formal health lending. The 1980 *World Bank Annual Report* declared the goal of ensuring basic access to core health services by 2000 but qualified that village workers needed more training, money would have to be allocated more effectively and staff recruitment and supply distribution needed improvement. Nevertheless, ‘[t]he Executive Directors agreed that emphasis should be placed on providing primary level health care to treat common, simple ailments; on preventive care instead of on curative medicine; on low-cost technologies in place of sophisticated hospitals and equipment; and on community participation in health care systems’.^32^ On the other hand, the 1980 *World Development Report* was tentative, arguing that ‘[t]he 1970s have thus witnessed the evolution of a much broader approach to health policy, including an emphasis on universal low-cost basic health care. But despite some successful experiments, “primary health care” is still more of a slogan than a nationwide reality in most developing countries. To change this is the greatest health challenge of the 1980s’.^33^

There was a background context to these debates about HFA’s potential. Throughout the 1970s, there were growing concerns for the mounting external debt crises in developing countries. Unpaid loans from post-war development projects built up due to overly abundant foreign aid in the absence of domestic savings.^34^ OPEC nations that accumulated ‘petrodollars’ following the 1970s oil embargoes had channelled them into American private banks, who then lent significant amounts to Latin American countries. Almost immediately after Alma Ata, Mexico declared in 1982 its inability to meet international repayments amounting to US$80 billion, shocking the world, included the Bank, which had never experienced debt of this scale in a developing country borrower.^35^ Despite periodic concerns expressed by the Mexican government and within the Bank throughout the 1970s, promising news of oil reserve discovery throughout the decade dimmed accurate economic monitoring and lending continued at an unsustainable rate to further the institution’s influence.^36^ Other Latin American countries soon followed, as Chile in 1982 and Peru in 1983 also declared balance of payments emergencies.^37^

In many ways, WHO tried to redefine HFA in the emerging debt crises. By December 1980, the Executive Board issued specific amendments on the Global Strategy for HFA by the Year 2000 to reflect the world economic situation, concluding that it could still be achieved by most nations ‘at no extra cost through the reshaping of their health systems as necessary and the judicious reallocation of existing resources accordingly’.^38^ Following this, in 1981, Mahler commissioned Lee Howard, former health director of USAID, to conduct a large-scale study resulting in the report ‘A New Look at Development Cooperation for Health’. Based on interviews with bilateral and multilateral donors, the premise that catalysed the study was that:

[i]n view of an uncertain world economic outlook for the coming decade, a healthy skepticism is often expressed in unofficial discussions regarding the prospect of achievement of such a goal in so short a time. Although there is emerging consensus on technical objectives and strategies to meet the challenge of HFA, there is less agreement on its economic feasibility within the next two decades. What then can realistically be done to mobilise resources both within and external to countries-in-need to achieve minimum access to health services and outcomes for the world’s majority? How is the bill to be paid? Who will pay?^39^

Howard’s first finding was that donors’ health assistance was highly dependent on recipient preferences. Even if health was declared a significant priority in international forums, countries usually requested health funding either for institutional medicine or when the investment would bring development to other sectors, such as agriculture, food production and education. Second, he found national health plans had not been substantially restructured towards the achievement of HFA by 2000, with most developing countries prioritising economic or military goals.^40^ Thus, Howard made a key recommendation that WHO play a coordinating role to counter the presence of multiple donors with wide-ranging and sometimes inconsistent agendas, as well as encourage country governments’ serious prioritisation of health.^41^ However, while the study itself was exhaustive, at over 600 pages, it strangely did not profile World Bank or speculate as to how WHO might effectively collaborate given the recession, despite it becoming the largest health lender at this time and a key node in the international financial system.^42^

Incorporating Howard’s recommendations, WHO’s re-conceptualisation of HFA was formalised in the Thirty-fourth World Health Assembly of May 1981. The Assembly encouraged member nations to develop stronger management systems, while promising to be considerate of resource mobilisation and rationalisation, issuing *Planning the Finances of the Health Sector* in 1983.^43^ Moreover, WHO would take responsibility for ensuring coordination in international health work, strengthening its original ‘unique constitutional role…acting as the directing and coordinating authority on international health work and ensuring technical cooperation between WHO and its Member States’.^44^ This renewed HFA strategy was expressed most vividly at a Financing Health Development seminar in 1982 in Manila, where Mahler gave a speech titled ‘Eighteen Years to go to Health for All’. Even in the recession, his belief in HFA remained unshaken.^45^ In fact, it was precisely because of the economic crises that non-costly basic primary health care had to be a priority:

Our Strategy is uncommon sense, and it takes uncommon courage to stand up for it. Moreover, what is the alternative, particularly in the face of economic realities? We cannot wait until even the most optimistic of medical conventionalists realises that if resources remain constant and technology becomes more and more costly, the breakdown point will soon be reached, and even fewer people will have access to health care.^46^

Mahler stressed in particular the member nations’ responsibilities to develop a coherent health plan appropriate to the context and build up infrastructure in order of priority, which should naturally begin with primary care: ‘You have to decide what activities if any among these programmes you require to develop your health system.’^47^ Finally, he warned ‘not look to WHO or any other international organisation for supranational salvation. Salvation will come from national action’.^48^

Meanwhile, new leadership in the early 1980s of Alden Clausen and Anne Kreuger signalled a change in the World Bank’s development paradigm. Appointed President in 1981, Clausen was well versed in commercial banking, once serving as Bank of America president and CEO, which had lent heavily to Latin American countries. Despite this, Clausen in his new post wrote to Mexican President López Portillo on 19 March 1982 that ‘the recent setback for the Mexican economy is bound to be transient, and we will be happy to be of assistance curing the consolidation process’.^49^ In addition, Anne Kreuger, appointed chief economist in 1982, was known for taking an aggressive stance towards the issue of trade and debt and famously coined the term ‘rent-seeking’ in a 1974 paper to describe inefficient interventionist public services.^50^

However, structural adjustment led by Clausen and Kreuger filtered slowly into the Bank’s first health loans, as the earliest projects from PHN’s establishment until 1986 were supportive of WHO’s HFA and primary care.^51^ As Mollie Fair has noted, between 1980 and 1986, health loans focused on broadening cost-effective basic healthcare access, which was partly due to client demand and HFA’s popularity.^52^ Thus, the 1981 Tunisian health and population project’s core objective was to expand primary health care.^53^ Kenya similarly in 1982 received an integrated rural health and family project loan, wherein:

[a] key element of primary health care, or of any health care system that attempts wide coverage at relatively low cost, is the use of community health workers (CHWs) with limited training to provide front-line services and to refer patients to rural health facilities and hospitals.^54^

Throughout the 1980s, it became clear to PHN staff that these primary care projects had serious shortcomings in practice. One point of contention was the romance of the village health worker. As Medcalf and Nunes have examined, WHO heavily centred the figure of the community health worker in HFA promotion, exemplified in the iconic Chinese barefoot doctors.^55^ Liese was one such sceptic, noting that the barefoot doctors themselves were not the only reason for disease control’s success in China, rather they were placed within an overall efficient and well-distributed infrastructure of anti-epidemic stations.^56^ Moreover, the Chinese public health system was undergoing financing reforms during the 1980s, which the Bank noted in its 1986 rural health and preventive medicine loan.^57^ This mixed financing approach included user charges, risk coverage and efficient use of private sector actors.^58^

## 3 The African Crisis 1985: Testing Health for All

It was ultimately the African crisis of 1985 that crystallised WHO and World Bank’s diverging views on health. Following Latin America, countries in Africa began facing mounting debt repayment issues.^59^ The Bank had been monitoring the situation since 1981, publishing *Accelerated Development in Sub-Saharan Africa* recommending structural adjustment policies, more popularly known as the Berg Report. The Berg was roundly criticised, even more so than the Bank’s actions in Latin America, for being particularly harsh on corruption and parastatals in African governments.^60^

Moreover, African countries faced continuous drought and famine throughout 1983 and 1984.^61^ The *New York Times* reported on 7 June 1983 that it was the worst food shortage faced in the region since the early 1970s, particularly in Ethiopia, Chad, Mozambique and Angola.^62^ In addition to structural adjustment, the Bank launched a Joint Programme of Action for sub-Saharan Africa in 1984, appealing for a total of $1 billion, and in 1985 approved a $3 million emergency food aid grant to be distributed by the World Food Program as quickly as possible.^63^ Sub-Saharan Africa assumed a centrality in World Bank’s programming from the mid-1980s onwards, with the Economic Development Institute expanding policy seminars with senior government officials regarding adjustment and numerous ‘Special Facilities’ for raising funds or hosting policy consultations regarding capacity building, agricultural research, small and medium-sized enterprises and non-governmental organisation (NGO) outreach.^64^ In the international health sphere, the African crisis’ dual nature as a humanitarian and a debt problem tested the logic of HFA, in particular its underlying basis in a New International Economic Order.^65^

Controversially, WHO decided to respond in a way that expressed HFA’s underlying principles for self-sufficiency and building up long-term resources over hands-on direct interventions. This was in part due to limited resources but also because it argued famine and drought were not particularly new disasters in the region. As with HFA, embedded in WHO’s actions was a critique of sudden outpourings of sympathy and charitable donations that would set up a ‘vicious cycle created by transient alleviation’.^66^ Actions focused less on food and refugee relief, since these were the remits of other agencies, and ‘the best way to recovery is to ensure that medium- and long-term measures of health infrastructure development are undertaken alongside emergency life-saving efforts’.^67^ Thus, WHO appealed to other members of the international community for help in the case of Angola or providing packages of oral rehydration salts in Botswana. In Burkina Faso, Gambia, Guinea, Lesotho, Malawi and Sierra Leone, WHO provided tetracycline tablets, yellow fever vaccine, health laboratory material and extra budgetary support for maternal and child health programmes, but also ‘a thorough updating of the country’s use of health resources in support of health-for-all policies through the health resource utilisation review (CRU) mechanism’.^68^

WHO’s focus on the long-term caused concern for the Bank. In a 15 February 1985 letter to Clausen, Mahler was defensive, arguing that they had a limited capacity to respond to an emergency such as the one currently ongoing in African nations:

We have just completed another session of our Executive Board during which anxiety was expressed over WHO’s contribution to the mitigation of the critical situation in Africa. I explained to the Board, and I believe this was well understood, that WHO’s role in direct emergency assistance is limited but that we have a major role in cooperating with affected countries in such a way that they not only extricate themselves from the immediate crisis but make genuine progress in terms of socio-economic development.^69^

By the Thirty-eighth World Health Assembly, in May 1985, a wide variety of views were expressed, including several calls for the serious implementation of NIEO.^70^ Mahler’s opening speech justified HFA’s focus on building capabilities over providing immediate emergency solutions:

At best it alleviates; at worst it subjugates. It can produce economic and social dependence that is certainly no less evil than dependence produced by drugs. There is always the danger that the self-cleansing, self-righteousness of giving to the unfortunate poor down there will blind the givers to the needs for more fundamental long-term solutions. I am afraid that danger is with us today.^71^

Delegates from severely affected African countries presented a range of views, neither wholeheartedly supporting nor roundly criticising HFA. The Ethiopian representative, the country where famine was most severe, stressed continued emergency relief from international agencies. Senegal and Zambia’s delegates similarly argued that the political instability in southern Africa would mean ‘health for some but not for all’.^72^ The Sudan representative related how it was now shouldering new responsibilities as a result of the crisis, but echoed Mahler’s views on self-sufficiency that ‘[w]e are resolved to depend in the first place upon our own efforts’.^73^

By the Thirty-ninth Assembly, member nations began seriously requesting HFA’s reassessment within the critical economic situation.^74^ The main critiques dealt with WHO’s understanding of how scarce resources should be allocated to the health sector. The Nigerian representative related that the stabilisation measures had demanded sacrifices on the slow path towards economic recovery, but the government was aggressively implementing measures to regulate parastatal enterprises, encourage compulsory savings schemes and push the agricultural sector, firmly adding that:

[t]he grim reality of the economic situation in Africa and the gloomy prospects for the future, at least in the short term, as described in document A39/4, pointed to the need to refrain from using scarce resources on wasteful and prestigious programmes and to concentrate on projects to benefit the silent majority, namely the deprived masses living in rural areas, as well as projects likely to generate internal and external revenue for the government. The depressed economic situation of the African countries was adversely affecting the health of their people and the orderly implementation of the health-for-all strategy.^75^

Other nations similarly requested clarifications regarding the relationship between development and health, as the Morocco representative asked WHO to explain in more precise terms what HFA after structural adjustment meant:

[t]he remedies suggested by various international economic and financial organisations to deal with the crisis were generally confined to reducing public consumption so as to decrease or at least stabilise the external debt. However, a reduction in public consumption also meant a reduction in health expenditure, since the latter was not always considered a priority. It was therefore the task of WHO to explain, through studies, the relationship between socioeconomic development, health promotion and health sector development in the various countries and regions of the world.^76^

WHO’s earlier efforts to consider financing after the Latin American crisis, such as the Howard report, became even more pronounced after 1985. The Seventy-seventh Session of the Executive Board acknowledged that ‘world economic prospects have deteriorated’, and that financial planning was the next step.^77^ In this reassessment, HFA failed because WHO had not properly costed the plans and health ministers had been reluctant to submit baseline information that would allow financial master plans, including alternative funding sources. WHO subsequently promised to promote seminars and studies on health economics and sector financing.

Meanwhile, the Bank’s loans shifted from primary care towards health sector reform, a stance it declared outwardly through the 1986 *Financing Health Services in Developing Countries* that recommended various cost recovery measures including the controversial user fees and insurance schemes.^78^ At the same time, staff were not wholly supportive of the Bank’s move towards health sector reforms. Nancy Birdsall, who held a number of senior PHN positions, including Policy and Research Division Chief, recalled that:

[b]y the mid-’80s, it was very problematic in the Bank, almost frustrating and annoying, to be working on sectoral issues, family behavior, the link to policy, education, health, even health financing, which is getting a little less…about program behavior and a little bit more macro….^79^

Similarly for Liese, it was difficult to believe the reforms were ‘not merely a whitewash’ to compensate for the considerable decline in development aid after the Cold War. The reforms also had contradictory impacts on the ground. In Indonesia, initially the hospital charges for patients were seen as practical. Patients unable to pay were exempt, and in the country’s village classification system, those destitute but needing care were known to administrators. A few years later, the charges had risen and were levied on all hospital goers. Doctors had to transfer the funds to the district officer, which were then used to finance public projects, such as road transport. Patients, even the poorest, were effectively funding the incomes of both the doctors and the government workers.^80^

Nevertheless, with WHO’s open acknowledgement that HFA needed reconceptualising, PHN expanded its advocacy for health financing throughout 1986. To express their renewed collaboration, WHO (represented by Ingar Bruggemann, Joshua Cohen and Andres Creese, among others) and PHN (John North, Nancy Birdsall and Anthony Measham) held an informal brainstorming meeting in Geneva in September 1986 with the fundamental acknowledgement that:

[h]uge disparities in the distribution of health services are exacerbated, favoring the advantaged at the expense of the disadvantaged: attempts to offer ‘free care to all’ often turn into ‘some care for a few and little for the rest’, and unfortunately ‘the rest’ are disproportionately poor.^81^

The two agreed HFA was weak on costing and ‘[f]acing the political and economic challenge to match our vision with the actual attainment of health for all – at national and international levels – may mean that promises to provide free health care have to be politically swallowed’.^82^ Moreover, PHN updated WHO on the IMF’s structural adjustment loans, as ‘The Effects of Adjustment Policies on Health and Nutritional Status’ was part of their agenda for discussion.^83^ Thus, financing – particularly cost recovery, mixed insurance schemes and the precise role of government in providing healthcare – became the crux of the two organisations’ dialogue leading into the era of AIDS.

At the same time, the September meeting was also a chance to think through the future course of their collaboration. One of the reasons was that the Bank had something to benefit from collaborating with WHO. PHN particularly wanted help in systematic collection of health data across countries. As Birdsall, who led the discussion, articulated, this was because they currently had no way of knowing whether their investments in health, nutrition and population were working and ‘the Bank is going to have a continuing interest in monitoring the effects of macroeconomic policy changes on health and other aspects of “living standards”’.^84^ USAID had been a pioneer in this kind of comprehensive global data system through its World Fertility Surveys (WFS), Demographic and Health Surveys (DHS) and Contraceptive Prevalence Surveys (CPS). PHN sought to do something similar, for which it deemed that ‘WHO ought to take the lead in mounting such a[n] effort’, while the Bank provided financial and technical assistance.^85^ WHO was still the premier technical expert on health and retained its potential to be a research coordinator, as PHN proposed ideas such as joint research in vaccine and drugs development and human reproduction.

Meanwhile, the Bank had quietly begun lending for AIDS, adding components to ongoing population, family health, disease control or sexually transmitted disease (STD) programmes in African countries as well as Brazil and Haiti, where the disease was considered most serious at the time.^86^ However, project details as described in 1987 indicated a reluctance to take charge independently of WHO regarding the biomedical and public health aspects of an unknown disease. The most detailed programmes were in Zaire, Zambia, Uganda and Burundi, wherein projects were as advanced as establishing surveillance systems, screening all blood donors, rolling out public education initiatives and even ‘[i]ntegration of AIDS control activities (e.g. blood screening, instrument sterilisation, case management, social support to families, counselling of patients) in the national health system’.^87^ In other countries, AIDS components were sparsely detailed. The Bank’s first involvement was in Ethiopia in April 1987 under the existing Family Health Project, though it ‘[did] not presently include AIDS components in a direct way’.^88^ Niger’s ongoing project was to be remolded to contain an AIDS aspect and Tanzania’s could potentially include condom distribution, though for both ‘[s]pecific activities remain to be determined’.

Naturally, financing and appropriate allocation of resources guided the Bank in its earliest approaches to AIDS, as it studied the disease’s economic consequences. Nevertheless, prevention of an infectious disease was still fundamentally a public good to be delivered at a national level. The Bank concluded that AIDS should be addressed by a core national disease control programme because this was an area wherein private for-profit actors would never involve themselves, particularly for the poor and marginalised. While in general, developing nations needed to reassess the place of the state in providing social and welfare services,

[u]nquestionably there are compelling reasons for government to remain a primary provider where private markets cannot suffice: for example, for disease control programs, for certain other preventive services, and for curative services in cases where private providers might never step in (remote, poor areas) or where a lengthy transition period will be required before private providers become well established.^89^

However, through cost-benefit rationalisation, the Bank concluded that parts of the AIDS response should be left to private providers, drawing from its earliest experiences in African countries. For example, blood screening was classified as a private good, while information, education and communication (IEC) would be a public good.^90^ In locations where there was a low chance of blood donors being seropositive, such as in Nigeria, it would cost an inefficient amount of government spending on blood testing as a universal service to screen and exclude that individual. At the rate of $2 per screening, if only one in 30 000 was likely to be positive, it would cost more than $60 000 to successfully identify that person through government blood testing. Publicly funded testing was therefore inefficient and should be conducted through unofficial private markets for transfused blood, which allowed at a small extra cost the service of having blood screened for HIV: ‘the high costs and low benefits from universal screening combined with the private good aspect of transfusions create an argument for a laissez-faire approach to transfusion’.^91^

## 4 The 1987 Collaboration on AIDS: Macroeconomic and Demographic Modelling

We do agree that it is critical to maintain a constructive working relationship between our units. We may have been remiss so far in not giving you a complete picture of our interests and needs in this area, as well as in coming to the area relatively late. However, my visit here was meant to partly remedy this, and it is unfortunate that I have not had the chance to discuss the issues with Dr. Carballo or you for more than a few minutes…. I understand that you are all extremely busy, and I therefore do not take this personally. For the future, however, in the interests of our working relationship, I would recommend some adjustments.^92^

As AIDS became an international crisis, the two agencies agreed to collaborate throughout 1987 to research the macroeconomic and demographic impact of AIDS in developing countries. Furthermore, the Bank’s need for WHO’s biomedical and epidemiological capabilities to create its desired global database for evaluating health projects became an opening for their cooperation in AIDS. Throughout 1987, PHN seconded to WHO Mead Over, a health economist, and Randy Bulatao, a demographer and consulting epidemiologist.^93^ Drawing from technical assistance missions throughout 1987 and 1988 in the various countries that had requested WHO or Bank assistance, Over was to investigate a computer-based model for estimating the cost-effectiveness of alternative AIDS programmes in sub-Saharan Africa which could be applied to any country.^94^ Bulatao was to work with James Chin, WHO’s chief of surveillance in the Forecasting and Impact Assessment Unit, to develop a demographic impact model.^95^

Unfortunately, there was a great deal of confusion from the very beginning. Bulatao was not able to meet Jonathan Mann, director of WHO’s Special Programme on AIDS (SPA), and his colleague Manuel Carballo, for more than a few minutes and Mann later sent a telegram to Anthony Measham requesting to delay Over’s Geneva visit as he wanted ‘clarification on the nature and scope of our collaborative relationship on AIDS’.^96^ When Bulatao did finally arrive in Geneva, most of WHO/SPA staff had been ‘too busy to see me for more than a few minutes and declined to discuss their objections’.^97^ Carballo managed to find some time to meet with him, but ‘mainly to object to some of my comments and to question my presence there’. At first, Bulatao thought WHO was not aware of the work on demographic modelling, though Birdsall had visited them again in May that year to discuss the Bank’s interests in demographic and economic research. He concluded that the reason for the lack of a proper reception might be that ‘[p]ossibly SPA/WHO was under the impression that my visit was solely to assist them, and had no connection to Bank work’.^98^

Thus, a significant portion of the collaboration happened through long and sometimes passive aggressive letters. As Bulatao communicated to Mann and Carballo in the absence of a face-to-face meeting, initially the Bank chose to ignore AIDS by not factoring it into its annual demographic projections of each country, which was a traditional practice due to its investment in population control.^99^ Though it had already been dealing with AIDS in a piecemeal fashion starting in 1986 by adding STD or AIDS components to existing health or population projects, AIDS was such a new issue that he made it clear the Bank wanted to collaborate with WHO largely because it did not understand how to deal with outbreaks of disease. However, AIDS was undeniably starting to impact its development work, meaning it would be ‘irresponsible for us to continue to ignore the phenomenon if there is something that can be done’.^100^

Mann, Carballo and WHO/SPA’s stand-offish absence was somewhat unprofessional, given that the collaboration had already been formally arranged. However, the attitude of the seconded PHN staff was also not conducive to a working partnership. Bulatao was defensive when explaining why the Bank acted without consulting WHO, while acknowledging that it was perhaps late to address AIDS and could have been more forthcoming about their interests. It had not intended to leave WHO out and an early draft was provided, the impression being that there were no reservations, but ‘[i]f you have now come to believe the Bank should not be doing any work on the demography of AIDS, Dr Measham and Mr Jamison will I am sure consider your arguments carefully, but will also have to take into account our own needs in the areas of demographic projection and economic work’.^101^ Bulatao simultaneously pressed WHO’s help to create the global database for evaluating health projects. Upon disclosing the Bank’s interests, he followed with a rather brusque assessment of WHO’s current usefulness, that ‘SPA/WHO cannot supply appropriate demographic statistics or an appropriate model for our demographic work, and does not intend to develop the capacity to do this or to directly support any institution that might provide such input’.^102^

Ultimately, the collaboration was not fruitful, as Bulatao reported to Dean Jamison, PHN chief, that WHO/SPA staff had ‘appeared reluctant to accept any Bank role in this area’ and ultimately advised that the Bank proceed on its own path.^103^ While WHO did not intend to assist the Bank with modelling, there was still potential for the organisation to serve as a source of epidemiological data, though Bulatao reported that he had ‘found nothing to indicate any institution with well-developed capacity in this area’.^104^ Thus, despite an attempt at multilateral collaboration amidst their increasing dialogue on financing after the African crisis of 1985, the Bank and WHO were not able to jointly address AIDS due to contrasting institutional ideologies expressed through individual personalities.

## 5 Parting Ways: WHO’s Response to Global AIDS

In the midst of its collaboration with the Bank, WHO had been confronting AIDS as the international authority on disease control. As Merson (who became director of WHO’s Global Programme on AIDS in 1990, taking over from Jonathan Mann) and Inrig have recently shown, Mahler was late to address AIDS as a serious issue, due to his commitment to HFA and belief that it was limited to the developed West.^105^ They show, with oral histories conducted in the early 2000s, that it was through the efforts of first Fakhry Assaad and later Jonathan Mann that WHO eventually came to form a human-rights-based strategy on AIDS: what was first called the Control Programme (CPA), then the Special Programme (SPA) and finally, the Global Programme on AIDS (GPA). AIDS was difficult to diagnose and treat, unlike diseases of previous WHO programmes, and it was only after concerted efforts by Assaad, director of the Division of Communicable Diseases, that Mahler agreed to establish a programme. Because of its complex manifestations and uncertainty around transmission and treatment, Assaad, Mann, Mahler and other WHO staff agreed independence and flexibility would be key to a successful response and the early AIDS programme was placed outside traditional WHO architecture, answerable only to the Director-General.^106^

As Cueto, Brown and Fee have noted, GPA under Mann was one of the most influential and well-funded programmes during a time of financial difficulty for WHO. In some ways, its strong emphasis on rights against discriminatory ‘containment’ practices responded to HFA’s underlying concerns regarding targeted intervention.^107^ At the same time, its extra budgetary funding at a time when regular contributions decreased made the programme answerable to donor objectives. As Merson and Inrig detail, WHO understood that they were in danger of creating precisely the kind of vertical, siloed and unintegrated programme that reflected Selective Primary Care’s objectives to control only the most serious diseases in developing countries.^108^ To counter this, Mann and Mahler promoted the idea of an integrated intersectoral response under the banner of health promotion, presenting this public health perspective at the International Conference on Health Promotion in Ottawa in November 1986.^109^ Moreover, Mann especially stressed the human rights aspects of the disease, inspired by early civil society responses in Western countries.^110^

The article’s final section situates Merson and Inrig’s arguments by exploring the continuities of WHO’s thinking on financing, health and development after the debt crises. There is no doubt that the organisation contributed a great deal to humane testing, blood banking, epidemiological surveillance, vaccine and treatment research and overall prevention systems around the world. However, the section suggests that WHO’s early AIDS activities continued to not fully resolve the concerns member nations expressed after the 1985 African crisis. As with its response in 1985, WHO bewilderingly used global AIDS as an opportunity to resolve what it perceived as persistent problems of aid coordination by attempting to control and coordinate donors, reasserting its position as the central agency on health.^111^ This, in combination with accommodating donor demands as part of extrabudgetary funding and the arrival of Hiroshi Nakajima in 1988, helps explain why GPA faced mounting criticisms by the early 1990s and many countries turned to the Bank’s International Development Association (IDA; concessional social sector loans designed for the societal impact of structural adjustment) for their first AIDS projects.

While WHO had been active in organising regional consultations specifically for the European and North American regions as early as 1983, the initial global AIDS response was formed after the first International Conference on Acquired Immunodeficiency Syndrome in Atlanta, Georgia in April 1985. Following these preliminary activities, a Global Strategy for HIV/AIDS was developed in what was then called the Control Programme on AIDS (CPA).^112^ CPA’s Global Strategy certainly paid more attention to the costliness of AIDS in ‘human and financial terms’. WHO accounted for the high treatment costs in developed countries, as a US hospital patient could expect to pay as much as $150 000. On the other hand, ‘[i]n developing countries, AIDS patient care depletes the already limited health care resources’.^113^ However, WHO/CPA did not extensively detail how AIDS programmes would be financed, arguing instead that ‘[t]he cost of this initial phase of a national programme clearly depends upon a variety of country-specific issues, especially regarding epidemiological surveillance and laboratory infrastructure’.^114^

During the era of CPA, WHO envisioned that its most appropriate role was as a global coordinator, mediator and advisor, facilitating communication where various bilateral and multilateral donors were active in one country. In doing so, WHO was trying to respond to HFA and NIEO concerns since the early 1980s of uncoordinated donor responses leading to harmful health effects, epitomised by Howard’s report. First, CPA would facilitate communication and information exchange between bilateral partners to coordinate the national programmes. Second, where there were gaps in the programme due to donor interests, it would provide medical supplies, consultants, evaluation exercises and other technical assistance. WHO’s role was more mediator, rather than controller, to ‘reduce competition with other agencies and help secure donor dollars to support national efforts’.^115^

By the time CPA had been re-established at the Fortieth World Health Assembly as the Special Programme on AIDS (SPA), WHO had become far more restrictive of donors. It designated a Joint Management Structure as well as a draft plan for ‘Comprehensive Coordination of Global and National AIDS Activities’.^116^ Comprehensive coordination expanded WHO’s global responsibility to ensure effectiveness amidst multiple donor agendas so that ‘[a]ll governmental, intergovernmental and non-governmental efforts, whether scientific, technical or financial, must be consistent with and supportive of WHO’s Global Strategy on AIDS as approved by the World Health Assembly’.^117^ Likewise, all country-level efforts had to be in line with a WHO-designed and managed National AIDS Plan, which would be consistent with the overarching Global Strategy, as well as primary health care within longer-term health goals: ‘[t]here should be a clear expression of the government’s acceptance of WHO’s responsibility for leadership and coordination’.^118^ The organisation also designated itself the single gatekeeper between recipient nations and donors for AIDS funding, conditional upon national governments wholly accepting WHO’s AIDS strategy.^119^ Merson argued that a large reason for this controlling response was that the US had drastically reduced its UN contributions. Building on this, this section suggests that WHO’s ambition was also a belated reaction to the international community’s scepticism over its actions in 1985.^120^

In spite of ongoing dialogue with the Bank, WHO subsequently sought partnerships with other UN agencies to target the development implications of AIDS. In December 1987, the WHO/UNDP Alliance to Combat AIDS announced that the ‘optimal solution is to combine the strengths of WHO as international leader in health policy and in scientific and technical matters related to health, and of UNDP as leader in socioeconomic development’.^121^ The drawing in of UNDP was deliberately done to showcase the UN family’s abilities to collaborate amidst funding difficulties.^122^ Finally, the echoes of the debt crises loomed, as the Alliance hoped to assist in better coordination of AIDS activities for the benefit of both the recipient nation and interested donors: ‘[t]he need for this has been reinforced by concern expressed by many countries about un-coordinated, ill-timed or inappropriate offers of external assistance’.^123^ However, the Alliance was stillborn as UNDP did not have the in-field staff capacity to adequately carry out the planned programmes.^124^ In addition, as Merson and Inrig have noted, there were interpersonal conflicts between Mann and Elizabeth Reid, founding director of UNDP’s HIV and Development Programme.^125^

Meanwhile, World Bank began to act increasingly independently of WHO, lending for larger-scale AIDS projects, such as the 1987 Population and Health Project to Burundi ($14 million) and the 1988 Northeast Endemic Diseases Control Project to Brazil ($109 million), which included a mass media campaign.^126^ As with HFA, PHN staff had increasing reservations about WHO’s global AIDS response. One was in regard to programme design, given how little was actually known about the disease. Anthony Measham, after a visit to WHO headquarters, observed that it did not appear to have a long-term vision for the next five- and ten-year periods, there were too many goals without prioritisation and it was unclear how contributing donors would receive updates on their investments. In addition, Mann was, in his view, overly optimistic about increased staff and budget, though ‘[w]e cautioned against undue haste and a “crash” mentality in building up the programme’.^127^ Some of the Bank’s concerns were gently communicated directly to WHO in more diplomatic tones than Bulatao’s. In an October 1987 telex, PHN Chief Jamison expressed to Mann that while the Bank was generally ‘sympathetic’ towards coordination, donors might feel inhibited by the requirements for consistency with the national plans.^128^ Jamison advised that WHO embed flexibility into their strategy, at the very least as a tactful and strategic gesture: ‘to express the possibility of desirability of such support, rather than an understanding that it will necessarily occur’.^129^

When Mahler’s term ended in 1988 without re-election, Hiroshi Nakajima became the new Director-General. If Mahler on PHC and Mann on AIDS had similarly ambitious social medicine approaches to health, preferring to tackle underlying conditions over treating manifestations, Nakajima was more a traditionalist and realist, seeking to address AIDS as a sexually transmitted disease than a problem of socioeconomic development and human rights. According to Merson, there were significant personal differences between Nakajima and Mann, leading to a fractured relationship ending with Mann’s resignation in 1990. WHO faced mounting criticisms into the early 1990s. As Fiona Godlee commented at the time, the organisation was criticised for its uncoordinated programmes and management structures.^130^ GPA had trouble maintaining extrabudgetary donor support and conflicting relations with other agencies, in particular UNDP, USAID and Ford Foundation. Thus, after extensive external review in 1992, GPA was dismantled and in its place, UNAIDS, a multiple donor co-sponsored institution including the Bank, was established in 1995.

In the meantime, the Bank moved from adding AIDS components to existing population, health or STD projects to providing large-scale IDA loans for AIDS only: $8.1 million to Congo in 1988, $84 million to India in 1992, $20.4 million to Chad in 1995 and $24.8 million to Indonesia in 1996.^131^ These early projects expressed the Bank’s thinking at the time regarding cost-effective public–private resource allocation, detailed earlier. For example, in India, the 1992 AIDS project was the first stand-alone health project after formal economic liberalisation and the 1987 Bank–Government of India health sector agreement.^132^ While the core technical public health aspects were WHO-design, as Assaad made frequent advisory trips to the country starting in 1986, when the first national case was identified, the 1992 Indian project reflected key aspects of the Bank’s views on cost-effective AIDS programmes: training and IEC delivery as public, blood safety as a mix of public and private banking centres and targeted health promotion by NGOs.^133^

Thus, after the African crisis of 1985 shifted the dialogue from HFA towards financing, the Bank and WHO began to dialogue even more closely. Financing concerns carried over into both agencies’ confrontations of AIDS in developing countries. However, WHO’s attempt to take top-down control of the global response, as well as conflicts between individual personalities, resulted in an uneasy division of labour until their co-sponsorship of UNAIDS: WHO covering the biomedical and public health aspects, with World Bank providing the funding and programme structure, often at the request of country governments, within an overarching costed health sector plan.

## 6 Conclusion

This article has related a story of collaboration and disagreement in global health financing during structural adjustment. Incorporating archival data from World Bank and focusing on its dynamics with WHO, it has contributed to historical understandings of the aftermath of the Alma Ata versus Selective Primary Healthcare debate in the late 1970s throughout the debt crises of the 1980s to the early era of global AIDS. While the historiography has examined US pressures on the UN system exerted via World Bank’s senior management or internal bureaucratic conflicts caused by Nakajima, the article has looked at individual collaborations and disagreements between WHO and PHN, particularly focusing on individuals such as Mahler, Mann, Liese, Birdsall and Bulatao, whose varying interpretations of the debt crises’ implications for health and development resulted in a tense collaboration.

A key empirical finding is how WHO selectively engaged with increasing pressures by the Bank and international community to consider financing, while trying to rationalise HFA’s logic amidst the emerging debt crises. Some of the reasons for HFA’s failure in the 1980s were circumstantial and beyond WHO’s control. Though Mahler commissioned Howard’s colossal project to re-conceptualise HFA within the global recession, it would have been impossible for an interview-based study by one individual to fully comprehend the complex landscape of post-war health-related aid, particularly when key actors like World Bank were not covered. At the same time, WHO’s adherence to HFA’s principles in key moments of expectation by the international community, such as the focus on self-reliance and long-term health systems development during the African crisis, did not help the cause of primary care.

The article has also pointed to how PHN situated itself at various points in the spectrum between Clausen and Kreuger’s structural adjustment, Walsh and Warren’s selective primary care and WHO’s social justice-oriented HFA. It has argued for histories of global health that distinguish World Bank’s functions as an international financing and development institution from its role as a key stakeholder in global health. It took some time for PHN to adapt to the health reforms dictated by structural adjustment following the Latin American crisis of the early 1980s. However, despite its greater lending capabilities, HFA did serve as a guiding principle for PHN throughout the early 1980s and the Bank continued to depend on WHO’s biomedical and epidemiological expertise in its first AIDS projects.

It has been nearly four decades since structural adjustment, a period broadly characterised as the Bank’s agenda to reform the economies and social sectors of developing countries towards integration within a capitalist global financial system. In recent years, there have been calls for more clarity in how the concept of ‘neoliberalism’ is deployed.^134^ With newly released archival documents, many available digitally, there is an opportunity to investigate various theories of historical change in global health, considering themes such as: attempts at mutually beneficial collaboration, well-meaning but impractical visions, insecurity directed towards a new and upcoming competitor, the need to accommodate senior management and the agendas of multiple actors.

## Footnotes

1.
World Health Organization (WHO), *Closing The Gap in a Generation: Health Equity Through Action on the Social Determinants of Health* (Geneva: Switzerland, 2008); WHO, *Primary Health Care: Now More Than Ever* (Geneva: Switzerland, 2008).

2.
WHO, ‘Impact of the Global Financial and Economic Crisis on Health: Statement by WHO Director-General Dr Margaret Chan’, 12 November 2008. Available online at https://www.who.int/mediacentre/news/statements/2008/s12/en/. Last accessed 24 October 2019.

3.
Theodore Brown, Marcos Cueto and Elizabeth Fee, ‘The World Health Organization and the Transition from “International” to “Global” Public Health’, *American Journal of Public Health*, 96, 1 (2006), 62–72; Marcos Cueto, Theodore M. Brown and Elizabeth Fee, *The World Health Organization: A History* (Cambridge: Cambridge University Press, 2019); Alexander Medcalf, Sanjoy Bhattacharya, Hooman Momen, Monica Saavedra and Margaret Jones (eds), *Health for All: The Journey to Universal Health Coverage* (Hyderabad: Orient Blackswan, 2015).10.2105/AJPH.2004.050831PMC147043416322464

4.

Jennifer Ruger , ‘The Changing Role of the World Bank in Global Health’, American Journal of Public Health, 95, 1 (2005), 60–70.1562386010.2105/AJPH.2004.042002PMC1449852

5.
Mohan Rao (ed.), *Disinvesting in Health: The World Bank’s Prescriptions for Health* (New Delhi: Sage Publications, 1999); Imrana Qadeer, Kasturi Sen and K.R. Nayar (eds), *Public Health and the Poverty of Reforms: The South Asian Predicament* (London and New Delhi: Sage Publications, 2001).

6.

Socrates Litsios , ‘The Long and Difficult Road to Alma-Ata: A Personal Reflection’, International Journal of Health Services, 32, 4 (2002), 709–732.1245612210.2190/RP8C-L5UB-4RAF-NRH2

7.
Alexander Medcalf and João Nunes, ‘Visualising Primary Health Care: World Health Organization Representations of Community Health Workers, 1970–89’, *Medical History*, 62, 4 (2018), 401–24; Jon Lidén, ‘The World Health Organization and Global Health Governance: Post-1990’, *Public Health*128, 2 (2014), 141–7.10.1017/mdh.2018.40PMC615864130191782

8.
Marcos Cueto, ‘The Origins of Primary Health Care and Selective Primary Health Care’, *American Journal of Public Health*, 94, 11 (2004), 1868; Randall Packard, *A History of Global Health: Interventions into the Lives of Other Peoples* (Baltimore: Johns Hopkins University Press, 2016), 285.10.2105/ajph.94.11.1864PMC144855315514221

9.

William Muraskin , The Politics of International Health: The Children’s Vaccine Initiative and the Struggle to Develop Vaccines for the Third World (Albany: State University of New York Press, 1998), 28.

10.
Cueto, Brown and Fee, *op. cit.* (note 3), 239–79.

11.

Nitsan Chorev , The World Health Organization between North and South (Ithaca: Cornell University Press, 2012), 130–140.

12.

Michael Merson and Stephen Inrig , The AIDS Pandemic: Searching for a Global Response (Switzerland: Springer International Publishing, 2018), 261–263.

13.

Jason L. Finkle and Barbara B. Crane , ‘The World Health Organization and the Population Issue: Organizational Values in the United Nations’, Population and Development Review, 2, 3/4 (1976), 367–393.

14.
Joseph Morgan Hodge, ‘Writing the History of Development (Part 2: Longer, Deeper, Wider)’, *Humanity*, 7, 1 (2016), 125–74; Lukas Engelmann, *Mapping AIDS: Visual Histories of an Enduring Epidemic* (Cambridge: Cambridge University Press, 2018), 216.

15.
WHO, *Constitution of the World Health Organization* (Geneva: World Health Organization, 1946); Sunil Amrith, *Decolonizing International Health: India and Southeast Asia, 1930–1965* (New York: Palgrave Macmillan, 2006); Sanjoy Bhattacharya, *Expunging Variola: The Control and Eradication of Smallpox* (Hyderabad: Orient Longman, 2006).

16.
Nils Gilman, ‘The New International Economic Order: A Reintroduction’, *Humanity*, 6, 1 (2015), 1; Natasa Miskovic, Harald Fischer-Tine and Nada Boskovska, *The Non-Aligned Movement and the Cold War: Delhi-Bandung-Belgrade* (London: Routledge, 2014).

17.
Packard, *op. cit.* (note 8), 229, 240.

18.
Chorev, *op. cit.* (note 11), 124.

19.
WHO, *Declaration of Alma Ata* (Geneva: WHO, 1978), Section IX, 3.

20.
WHO, *Thirty-Second World Health Assembly, Geneva, 7–25 May 1979* (Geneva: WHO IRIS), 21.

21.
Gilman, *op. cit.* (note 16), 1.

22.
Richard Jolly, ‘Adjustment with a Human Face: A UNICEF Record and Perspective in the 1980s’, *World Development*, 19, 12 (1991), 1809; Marcos Cueto, ‘The Origins of Primary Health Care and Selective Primary Health Care’, *American Journal of Public Health*, 94, 11 (2004), 1868.10.2105/ajph.94.11.1864PMC144855315514221

23.

Julia Walsh and Kenneth Warren , ‘Selective Primary Health Care: An Interim Strategy for Disease Control in Developing Countries’, New England Journal of Medicine, 301, 18 (1979), 967–974.11483010.1056/NEJM197911013011804

24.

*Ibid*., 967.

25.
Transcript of interview with Bernhard H. Liese by Charles Ziegler, 9 and 17 April 2008 (Washington, DC: World Bank, 2008), 2. Available online at http://documents.worldbank.org/curated/en/185691468328219018/Transcript-of-oral-history-interview-with-Bernhard-H-Liese-held-on-April-9-and-17-2008. Last accessed 20 October 2019.

26.

*Ibid.*, 14.

27.

*Ibid.*, 16.

28.
Mollie Fair, ‘From Population Lending to HNP Results: The Evolution of the World Bank’s Strategies in Health, Nutrition and Population’, IEG Working Paper 2008/3, World Bank Open Knowledge Repository. Available online at https://openknowledge.worldbank.org/bitstream/handle/10986/6406/432090NWP0From1Box0327352B01PUBLIC1.pdf;sequence=1. Last accessed 22 December 2019.

29.
Transcript of interview with James Lee by Bogomir Chokel, 4 April 1985 (Washington, DC: World Bank, 1992), 12–15. Available online at https://oralhistory.worldbank.org/transcripts/transcript-oral-history-interview-dr-james-lee-held-april-4-1985. Last accessed 17 October 2019.

30.
Liese, *op. cit.* (note 25), 14.

31.
Fair, *op. cit.* (note 28), 4.

32.
World Bank (henceforth WB), *World Development Report, 1980* (Washington, DC: World Bank, 1980), 71.

33.

*Ibid.*, 53.

34.
Stephen S. Golub, ‘The Political Economy of the Latin American Debt Crisis: Review of Various Works’, *Latin American Research Review*, 26, 1 (1991), 175–215; Syed Nawab Haider Naqvi, ‘The Balance-of-Payments Problem in Developing Countries’, *The Pakistan Development Review*, 12, 3 (1973), 259–72.

35.
Martin Honeywell, ‘The world debt crisis’, in Latin America Bureau, *The Poverty Brokers: The IMF and Latin America* (Latin America Bureau (Research and Action): Great Britain, 1983), Folder: 1213219, World Bank Group Digital Archives, 1. Available online at http://pubdocs.worldbank.org/en/532231486658669159/wbg-archives-1213219.pdf. Last accessed 30 December 2019.

36.

Devesh Kapur , John P. Lewis and Richard Webb , The World Bank: Its First Half Century, Vol. 1 (Washington DC: World Bank, 1997), 498–499.

37.

Manuel Pastor , ‘Latin America, the Debt Crisis, and the International Monetary Fund’, Latin American Perspectives, 16, 1 (1989), 79–110.

38.
WHO, ‘Health for All by the Year 2000 – Global Strategy: Specific Amendments Made by the Programme Committee to Document EB67/PC/WP3’, 8 December 1980, Sixty-Seventh Session of Executive Board, WHO IRIS, 2.

39.

Lee Howard , A New Look at Development Cooperation for Health: A Study of Official Donor Policies, Programmes, and Perspectives in Support of Health for All by the Year 2000 (Geneva: WHO, 1981), xvii.

40.
Cueto, Brown and Fee, *op. cit.* (note 3), 190.

41.
Howard, *op. cit*. (note 39), 10, 343.

42.

*Ibid.*, 7.

43.
WHO, ‘Regional Strategy for Health for All by the Year 2000: Financial Needs of the Strategy, International Flow of Resources’, 7 July 1982, WPR/RC33/9, WHO IRIS, 7; E.P. Mach and B. Abel-Smith, *Planning the Finances of the Health Sector: A Manual for Developing Countries* (Geneva: WHO, 1983).

44.
WHO, *Thirty-fourth World Health Assembly* (Geneva: WHO, 1981), 24.

45.
WHO, ‘Report: Seminar on Financing Health Development in the Western Pacific Region, Manila’, 31 May to 4 June 1982, Regional Office for the Western Pacific, WHO IRIS, 1; ‘Message by Dr H. Mahler, Director-General of the World Health Organization to the Thirty-third Session of the Regional Committee for the Western Pacific: Eighteen Years to go to Health for All, Manila’, 20–24 September 1982, WPR/RC33/DIV/2, WHO IRIS, 1–2.

46.

*Ibid.*, 2.

47.

*Ibid.*, 5.

48.

*Ibid.*, 3.

49.
Alden W. Clausen to José López Portillo, 19 March 1982, qtd. in Kapur, Lewis and Webb, *op. cit.* (note 36), 603.

50.

Anne Kreuger , ‘The Political Economy of the Rent-Seeking Society’, The American Economic Review, 64, 3 (1974), 291–303.

51.

Marlee Tichenor and Devi Sridhar , ‘University Health Coverage, Health Systems Strengthening, and the World Bank’, The BMJ, 358 (2017), j3347.2886013810.1136/bmj.j3347PMC5594415

52.
Fair, *op. cit.* (note 28), 5.

53.
WB, ‘Tunisia Staff Appraisal of a Health and Population Project’, 6 May 1981, Report No. 3204-TUN (Washington, DC: World Bank, 1981), 2, 36, 61.

54.
WB, ‘Kenya: Staff Appraisal of an Integrated Rural Health and Family Planning Project’, 6 May 1981, Report No. 3409-KE (Washington, DC: World Bank, 1982), 32.

55.
Medcalf and Nunes, *op. cit.* (note 7), 413.

56.
Liese, *op. cit.* (note 25), 13.

57.
X. Liu and A. Mills, ‘Financing Reforms of Public Health Services in China: Lessons for Other Nations’, *Social Science & Medicine*, 54, 11 (2002), 1691–8; Ian G. Cook and Trevor J.B. Dummer, ‘Changing Health in China: Re-evaluating the Epidemiological Transition Model’, *Health Policy*, 67 (2004), 329–43.10.1016/s0277-9536(01)00337-912113451

58.
WB, ‘Staff Appraisal Report China Rural Health and Preventive Medicine Project’, 27 May 1986, Report No. 5876-CHA (Washington, DC: World Bank, 1986), 1; WB, *Financing Health Services in Developing Countries: An Agenda for Reform* (Washington, DC: World Bank, 1986), 72.

59.

Henry F. Jackson , ‘The African Crisis: Drought and Debt’, Foreign Affairs, 63, 5 (1985), 1081–1094.

60.
Elliot Berg, *Accelerated Development in Sub-Saharan Africa: An Agenda for Action* (Washington, DC: World Bank, 1981); Giovanni Arrighi, ‘The African Crisis: World Systemic and Regional Aspects’, *New Left Review*, 15 (2002), 5–36; Kapur, Lewis and Webb, *op. cit.* (note 36), 719.

61.
Kapur, Lewis and Webb, *op. cit.* (note 36), 747.

62.
Bernard Weinraub, ‘Famine in Africa is Called Worst in a Decade’, *The New York Times*, 7 June 1983.

63.
Ibrahim Shihata, ‘The Power of the Bank to Make Grants’, 15 February 1985, File: 30158262 WHO, Vol. 7, World Bank Group Archives [henceforth: WBG].

64.
Kapur, Lewis and Webb, *op. cit.* (note 36), 731–2.

65.
Yves Beigbeder, Mahyar Nashat, Marie-Antoinette Orsini and Jean-Francois Tiercy, *The World Health Organization* (Boston: Martinus Nijhoff Publishers, 1998); Kelley Lee, *World Health Organization* (London: Routledge, 2008).

66.
World Health Assembly, ‘Collaboration within the United Nations System: Emergency Health and Medical Assistance to Drought-Stricken and Famine-Affected Countries in Africa, Report by the Director General’, 2 May 1985, A38/16, WHO IRIS, 3.

67.

*Ibid.*, 3.

68.

*Ibid.*, 3.

69.
H. Mahler to A.W. Clausen, 15 February 1985, File: Clausen’s WHO, Box: Clausen Papers-WHO Correspondences, Vol. 1, WBG, 1.

70.
However, the Meeting of Nonaligned Health Ministers in New Delhi reaffirmed commitment to HFA by 2000, with a letter of support stressing technical cooperation amongst developing countries, though it did not articulate a stance on the economic crises. ‘Communication from the Delegation of India Letter dated 8 May 1985 addressed to the President of the Thirty-eighth World Health Assembly’, 10 May 1985, A38/INF.DOC./11, WHO IRIS.

71.
Thirty-eighth World Health Assembly, ‘Verbatim Records of Plenary Meetings Reports of Committees’, 6–20 May 1985, 16.

72.

*Ibid.*, 213.

73.

*Ibid.*, 89.

74.
WHO, ‘Repercussions of the World Economic Situation: Report by the Director-General’, 10 December 1985, EB77/INF.DOC./2, WHO IRIS, 1.

75.
Thirty-ninth World Health Assembly, ‘Committee A: Provisional Summary Record of the Fourth Meeting, Palais des Nations, Geneva’, 10 May 1986, A39/A/SR/4, WHO IRIS, 2.

76.

*Ibid.*, 3.

77.
WHO, ‘Planning of the Finances of Health for All: Economic Strategies to Support the Strategy for Health for All, Report by the Director-General’, 4 November 1985, File: 30158265 WHO, Vol. 2, WBG, 2.

78.
PHN in 1987 was also decentralised into a division of the Population and Human Resources Department. WB, *Financing Health Services*, *op. cit.* (note 58).

79.
Transcript of interview with Nancy Birdsall by John Lewis, Richard Webb and Devesh Kapur, 15 November 1989 (Washington, DC: World Bank, 1989), 2–4. Available online at http://documents.worldbank.org/curated/en/906301468157524104/Notes-of-interview-with-Nancy-Birdsall-held-on-November-15-1989. Last accessed 20 October 2019.

80.
Liese, *op. cit.* (note 25), 29.

81.
‘Financing Health Services: PHN Draft Documents’, May 1986, File: 1104244 WHO, Vol. 1, WBG, 8.

82.
‘Jointly Agreed Annotated Agenda World Bank/World Health Organization Meeting on Health Policy and Collaboration, 3–4 September 1986, Geneva’, 1986, File: 1104244 WHO, Vol. 1, WBG, 2.

83.
‘World Bank/World Health Organization Meeting on Health Policy and Collaboration, 3–4 September 1986, Geneva’, 1986, File: 1104244 WHO, Vol. 1, WBG, 3.

84.
Nancy Birdsall to John North, cc: Paqueo, Bulatao, Hill, Barnum, Measham, 10 July 1986, File: 1104244 WHO, Vol. 1, WBG.

85.

*Ibid*.

86.
Callie Boucher and Lisa Pachter, ‘AIDS: The Bank’s Relations with WHO’s GPA and its own Policies’, 6 April 1987, File: 30158262 WHO, Vol. 7, WBG.

87.

*Ibid.*, 3.

88.
‘AIDS Components in Bank Projects’, 31 March 1988, File: 30158262 WHO, Vol. 7, WBG, 2.

89.
WB, *Financing Health Services*, *op. cit.* (note 58), 6.

90.
Mead Over to Dean Jamison, ‘Attachment II: Issues in Resource Allocation to AIDS Treatment, Prevention and Control’, 19 August 1987, File: 30158262 WHO, Vol. 7, WBG, 5.

91.

*Ibid.*, 5–6.

92.
R.A. Bulatao to Dr J. Mann, ‘Subject: Rationale for the Bank’s Interest in Studying Aspects of the AIDS Pandemic’, 27 June 1987, File: 30117078 AIDS-WHO-Bank Cooperation, WBG, 2–3.

93.
Mead Over to Dean Jamison, ‘Collaboration with WHO Special Programme on AIDS: Back-to-Office Report’, 19 August 1987, File: 30158262 WHO, Vol. 7, WBG.

94.
Mead Over to Nancy Birdsall and Dean Jamison, ‘Subject: Cooperation between the Bank and WHO on Analysis of Economic Impact of AIDS’, 4 June 1987, File: 30117078 AIDS-WHO-Bank Cooperation, WBG, 1–2.

95.
Dr J. Mann, ‘Attachment I: Note for the Record on a Conversation on 14 July 1987 Between Myself and Drs Anthony Measham, Mead Over and Dean Jamison of the World Bank’, 1987, File: 30158262 WHO, Vol. 7, WBG; James Chin, *The AIDS Pandemic: The Collision of Epidemiology with Political Correctness* (Oxford and Seattle: Radcliffe Publishing, 2007).

96.
Dr J. Mann to Dr A. Measham, 26 June 1987, File: 30117078 WHO, Vol. 7, WBG.

97.
R.A. Bulatao to Dean Jamison, ‘Subject: Visit to WHO Special Programme on AIDS (SPA/WHO), 18–30 June 1987: Back-to-office Report’, File: 30117078 AIDS-WHO-Bank Cooperation, WBG, 1.

98.

*Ibid.*, 3.

99.
Bulatao to Mann, ‘Rationale’, *op. cit.* (note 92), 2–3.

100.

*Ibid.*, 1.

101.

*Ibid.*, 1–2.

102.

*Ibid.*, 1.

103.
Bulatao to Jamison, ‘WHO visit’, *op. cit.* (note 97), 1.

104.

*Ibid.*, 2.

105.
Merson and Inrig, *op. cit.* (note 12), 19.

106.

*Ibid.*, 50–1.

107.
Cueto, Brown and Fee, *op. cit.* (note 3), 210.

108.
Merson and Inrig, *op. cit.* (note 12), 55–6.

109.
Cueto, Brown and Fee, *op. cit.* (note 3), 199.

110.

Elizabeth Fee and Manon Parry , ‘Jonathan Mann, HIV/AIDS, and Human Rights’, Journal of Public Health Policy, 29, 1 (2008), 54–71.1836801910.1057/palgrave.jphp.3200160

111.
Packard, *op. cit.* (note 8), 285; Merson and Inrig, *op. cit.* (note 12), 20.

112.
Control Programme on AIDS, *Global WHO Strategy for the Prevention and Control of Acquired Immunodeficiency Syndrome: Projected Needs for 1986–1987* (Geneva: WHO, 1986), 5.

113.

*Ibid.*, 3.

114.

*Ibid.*, 9.

115.
Merson and Inrig, *op. cit.* (note 12), 34.

116.
‘WHO: Special Programme on AIDS, Comprehensive Coordination of Global and National AIDS Activities’ and ‘Draft Proposal WHO SPA Joint Management Structure’, 30 September 1987, Series II: AIDS Background Files, Box 10, FA717, Ford Foundation records, Rockefeller Archive Center.

117.
‘Comprehensive Coordination of Global and National Activities’, 1, 10.

118.

*Ibid.*, 5.

119.
‘[O]nce the National AIDS Plan is finalised, approved by the government and sanctioned by WHO, WHO/SPA will field a negotiating team to support the government in convening a meeting for the purpose of mobilising resources for the Plan. Following this meeting, certain development agencies willing to support one or more components of the Plan, may offer to field teams to elaborate more detailed proposals.’ *Ibid.*, 4.

120.
Merson and Inrig, *op. cit.* (note 12), 37.

121.
‘WHO/UNDP Alliance to Combat AIDS’, 19 December 1987, WHO Executive Board, 81st Session, EB81/INF.DOC.3, WHO IRIS, 1; Merson and Inrig, *op. cit.* (note 12), 78–81.

122.
WHO/UNDP, ‘Press Briefing by WHO/UNDP’, 29 March 1988, DEB/1766, File: WHO World AIDS Day [S-1051-24-8], UN Archives, New York, 1–2.

123.
‘WHO/UNDP Alliance’, *op. cit.* (note 108), 1.

124.
Governing Council of the United Nations Development Programme, ‘HIV, AIDS and Development: HIV and Development National Professional Officers, Report of the Administrator’, 15–18 February 1994, DP/1994/5, UN Archives, New York, 2.

125.
Merson and Inrig, *op. cit.* (note 12), 270.

126.
WB, ‘Staff Appraisal Report: Burundi Population and Health Project’, 16 November 1987, Report No. 6829-BU (Washington, DC: World Bank, 1987); WB, ‘Staff Appraisal Report: Brazil Northeast Endemic Disease Control Project’, 19 January 1988, Report No. 6837-BR (Washington, DC: World Bank, 1988).

127.
Anthony R. Measham to John D. North, ‘Subject: Visits to WHO/Geneva and LSHTM, January 5–13, 1987: Back to Office Report’, 20 January 1987, File: 30158262 WHO, Vol. 7, WBG.

128.
Dean Jamison to Mrs I. Bruggemann and Dr J. Mann, ‘Subject: World Bank Comments on Draft Management and Coordination Documents for Special Programme on AIDS (SPA)’, 23 October 1987, File: 30117078 AIDS-WHO-Bank Cooperation, WBG.

129.

*Ibid.*, 3–4.

130.

Fiona Godlee , ‘The World Health Organisation: WHO in crisis’, BMJ, 309 (1994), 1424.808693810.1136/bmj.309.6954.553PMC2541415

131.
WB, ‘Projects List’, *Projects and Operations* Available online at https://projects.worldbank.org/en/projects-operations/projects-list?searchTerm=AIDS. Last accessed 26 October 2019.

132.
WB, *Case Study of World Bank Activities in the Health Sector in India* (Washington, DC: World Bank, 1999), 8.

133.
Merson and Inrig, *op. cit.* (note 12), 25; Directorate General of Health Services, Ministry of Health and Family Welfare and World Health Organization, ‘Medium-Term Plan for the Prevention and Control of AIDS in India, 1990–1992’, February 1990, RR-P IND.M 406, archives of the MAMTA Health Institute for Mother and Child, New Delhi; WB, *Staff Appraisal Report: India National AIDS Control Project*, 9 March 1992, Report No. 10165-IN (Washington, DC: World Bank, 1992), viii, ix, 3–5, 7, 11–13, 16–21.

134.
Kirsten Bell and Judith Green, ‘On the Perils of Invoking Neoliberalism in Public Health Critique’, *Critical Public Health*, 26, 3 (2016), 239–43; Ted Schrecker, ‘“Neoliberal Epidemics” and Public Health: Sometimes the World is Less Complicated than it Appears’, *Critical Public Health*, 26, 5 (2016), 477–80; James Ferguson, ‘The Uses of Neoliberalism’, *Antipode*, 41, 11 (2009), 166–84.

